# Supplementary material for: Modeling glioblastoma heterogeneity as a dynamic network of cell states
Source: Mol Syst Biol. 2021 Sep 16;17(9):e10105. doi: 10.15252/msb.202010105 (PMC8444284; doi:10.15252/msb.202010105)
Supplement: Supplementary file 6 — Source Data for Figure 5 [file MSB-17-e10105-s004.zip › Figure5A_sourcedata/GSEA_3071/hallmarks_stateI.GseaPreranked.1623742531165/HALLMARK_PEROXISOME.html]

Details for gene set HALLMARK\_PEROXISOME[GSEA]

|  || Dataset | state13071 |
| Phenotype | NoPhenotypeAvailable |
| Upregulated in class | na\_neg |
| GeneSet | HALLMARK\_PEROXISOME |
| Enrichment Score (ES) | -0.27243426 |
| Normalized Enrichment Score (NES) | -0.97396237 |
| Nominal p-value | 0.48381603 |
| FDR q-value | 0.61666656 |
| FWER p-Value | 1.0 |
Table: GSEA Results Summary

  

Fig 1: Enrichment plot: HALLMARK\_PEROXISOME      
 Profile of the Running ES Score & Positions of GeneSet Members on the Rank Ordered List

  

| PROBE | GENE SYMBOL | GENE\_TITLE | RANK IN GENE LIST | RANK METRIC SCORE | RUNNING ES | CORE ENRICHMENT || 1 | SEMA3C |  |  | 402 | 0.586 | -0.0588 | No |
| 2 | PRDX1 |  |  | 447 | 0.549 | -0.0186 | No |
| 3 | PEX11A |  |  | 639 | 0.439 | -0.0311 | No |
| 4 | CLN8 |  |  | 718 | 0.405 | -0.0145 | No |
| 5 | HSD17B11 |  |  | 779 | 0.389 | 0.0057 | No |
| 6 | TSPO |  |  | 826 | 0.373 | 0.0284 | No |
| 7 | SCP2 |  |  | 868 | 0.361 | 0.0513 | No |
| 8 | GSTK1 |  |  | 1113 | 0.304 | 0.0107 | No |
| 9 | IDH2 |  |  | 1324 | 0.264 | -0.0241 | No |
| 10 | MSH2 |  |  | 1438 | -0.252 | -0.0322 | No |
| 11 | CTPS1 |  |  | 1540 | -0.261 | -0.0360 | No |
| 12 | ACAA1 |  |  | 1862 | -0.300 | -0.0991 | No |
| 13 | HRAS |  |  | 2276 | -0.367 | -0.1821 | No |
| 14 | ERCC1 |  |  | 2397 | -0.392 | -0.1788 | No |
| 15 | YWHAH |  |  | 2725 | -0.479 | -0.2264 | Yes |
| 16 | TOP2A |  |  | 2842 | -0.517 | -0.2100 | Yes |
| 17 | SMARCC1 |  |  | 2973 | -0.584 | -0.1911 | Yes |
| 18 | CNBP |  |  | 3113 | -0.686 | -0.1649 | Yes |
| 19 | SLC25A4 |  |  | 3316 | -0.953 | -0.1312 | Yes |
| 20 | CADM1 |  |  | 3485 | -1.943 | 0.0074 | Yes |
Table: GSEA details [plain text format]

  

Fig 2: HALLMARK\_PEROXISOME: Random ES distribution      
 Gene set null distribution of ES for **HALLMARK\_PEROXISOME**
